# A Comprehensive Study of Pristine and Calcined f-MWCNTs Functionalized by Nitrogen-Containing Functional Groups

**DOI:** 10.3390/ma15030977

**Published:** 2022-01-27

**Authors:** Anna Bajorek, Bogumiła Szostak, Mateusz Dulski, Jean-Marc Greneche, Sabina Lewińska, Barbara Liszka, Mirosława Pawlyta, Anna Ślawska-Waniewska

**Affiliations:** 1 A. Chełkowski Institute of Physics, University of Silesia in Katowice, 75 Pułku Piechoty 1A, 41-500 Chorzów, Poland; szostakbogumila@gmail.com; 2 Silesian Center for Education and Interdisciplinary Research, University of Silesia in Katowice, 75 Pułku Piechoty 1A, 41-500 Chorzów, Poland; mateusz.dulski@us.edu.pl; 3 Institute of Materials Science, University of Silesia in Katowice, 75 Pułku Piechoty 1A, 41-500 Chorzów, Poland; 4 Institut des Molécules et Matériaux du Mans UMR CNRS 6283, Le Mans Université, Avenue Olivier Messiaen, CEDEX 9, 72085 Le Mans, France; jean-marc.greneche@univ-lemans.fr; 5 Institute of Physics, Polish Academy of Sciences, Al. Lotników 32/46, 02-668 Warsaw, Poland; lewinska@ifpan.edu.pl (S.L.); slaws@ifpan.edu.pl (A.Ś.-W.); 6 Faculty of Natural Sciences, University of Silesia in Katowice, Będzińska 60, 41-200 Sosnowiec, Poland; barbara.liszka@us.edu.pl; 7 Materials Research Laboratory, Institute of Engineering Materials and Biomaterials, Silesian University of Technology, Konarskiego 18A, 44-100 Gliwice, Poland; miroslawa.pawlyta@polsl.pl

**Keywords:** carbon nanotubes, structural properties, magnetic properties, electronic structure

## Abstract

We present the study of pristine and calcined f-MWCNTs functionalized by nitrogen-containing functional groups. We focus on the structural and microstructural modification tuned by the previous annealing. However, our primary goal was to analyze the electronic structure and magnetic properties in relation to the structural properties using a multi-technique approach. The studies carried out by X-ray diffraction, XPS, and ^57^Fe Mössbauer spectrometry revealed the presence of γ-Fe nanoparticles, Fe_3_C, and α-FeOOH as catalyst residues. XPS analysis based on the deconvolution of core level lines confirmed the presence of various nitrogen-based functional groups due to the purification and functionalization process of the nanotubes. The annealing procedure leads to a structural modification mainly associated with removing surface impurities as purification residues. Magnetic studies confirmed a significant contribution of Fe_3_C as evidenced by a Curie temperature estimated at *T*_C_ = 452 ± 15 K. A slight change in magnetic properties upon annealing was revealed. The detailed studies performed on nanotubes are extremely important for the further synthesis of composite materials based on f-MWCNTs.

## 1. Introduction

Carbon nanotubes (CNTs) were intensively studied immediately after their discovery by Suimo Ijima in 1990 [1]. He demonstrated the presence of nanocylindrical forms of pure sp^2^ carbon having several micrometres long and several nanometers in diameter [1,2,3]. Thus, CNTs can be considered as a hexagonal array of carbon atoms wound in a hollow cylinder in two ways: single-walled nanotubes (SWCNTs) and multi-walled (MWCNTs) nanotubes [1]. These materials are characterized in particular by (i) outstanding mechanical and structural properties; (ii) good surface to area ratio; (iii) unique electrical conductivity; (iv) chemical and thermal stability, making them attractive components for nanohybrids [4,5,6,7,8,9,10,11,12,13,14,15]. Nevertheless, CNTs, usually in their pristine form, tend to aggregate by forming agglomerates combined with van der Waals interactions [16]. Because pristine CNTs are hydrophobic and chemically inert, their functionalization with various suitable organic groups leads to new activities on the CNT surface for nanoparticle grafting, which enhances their hydrophilicity and dispersibility in several solvents [17,18,19,20,21,22,23,24,25,26]. Understanding the impact of the functionalization type on the various properties of CNTs is one of the key factors for their potential future applications. Keeping in mind the development of nanotechnology, the electronic and magnetic properties of CNTs could be exploited, for example, in the design and synthesis of nanocomposite materials [4,5,6,7,8,9,10,11,12,13,14,15]. In general, MWCNTs are promising candidates for building blocks to build nanocomposites. However, it should be emphasized that in describing the properties of these materials, the iron-like species embedded in MWCNTs must be considered, as the Fe catalyst precursor formed in situ is the active centre for carbon filament formation [27,28,29,30,31,32,33,34,35,36,37,38,39]. Usually, α-Fe, γ-Fe and Fe_3_C phases have been detected in Fe-filled CNT, but their relative content is strictly dependent on the synthesis procedure and post-treatment [27,28,29,30,31,32,33,34,35,36,37,38,39]. Baker et al. [27] demonstrated the detection of several iron carbides during the formation of carbon filaments. In contrast, based on X-ray diffraction analysis, Sacco et al. [28] revealed that the formation of Fe_3_C is responsible for the initial growth of carbon filaments and α-Fe as a product of heating in a gas mixture containing carbon precursors. The presence of several iron carbides as intermediates in filament growth has also been proposed by Geus et al. [29,30]. However, the detailed description of the nature of the iron precursor in CNTs is still a challenging task, and the use of multi-technique characterization to solve this problem is highly demanded. Schaper et al. [31] reported in situ electron microscopy studies demonstrating the role of cementite as an intermediate in the catalytic formation of nanotubes by promoting the successive segregation of new well-ordered graphene layers on its surface.

A promising tool for analyzing iron species in MWCNTs is ^57^Fe Mössbauer spectrometry, which allows the analysis of the different valence and spin states of Fe forms. So far, using this method, it has been shown that traces of Fe-based catalyst can be easily deposited inside MWCNTs during their synthesis and that iron aggregates can be reduced by almost 90% by the oxidation process [34,37,38,39,40]. However, the functionalization of carboxylated carbon nanotubes by fabricating CNT ammonium salt does not remove more Fe impurities but only changes the iron states. In pristine MWCNTs, the main component is Fe_3_C, transformed into ferrihydrites during the oxidation process. In addition, treatment with NH_3_ leads to further oxidation of the cementite, where half of the Fe_3_C is transformed into hydrated superparamagnetic Fe_2_O_3_ particles. However, the relative content of α-Fe in the iron grains embedded in the CNT samples remains relatively unchanged [34,38,39,40]. Prados et al. [36], based on Mössbauer analysis and hysteresis loops, claimed that the Fe catalyst precursors present in nanotubes consisted of a core of α-Fe surrounded by layers of γ-Fe, Fe_3_C, and carbon.

In view of the ongoing discussion on the form of Fe as a catalyst precursor in MWCNTs, in the following paper, we have focused on a comprehensive study of pristine and calcined f-MWCNTs functionalized with COONH_4_ groups. We present a detailed chemical and structural analysis of the Fe nanoparticles embedded in the nanotubes, combined with the analysis of the magnetic properties. We discussed the effect of calcination on the studied properties. The analysis we have performed will subsequently be effective in the ongoing synthesis of spinel ferrites nanoparticles/carbon nanotubes (SF-NPs/CNTs) nanocomposites targeting the synergic effect that could extend their potential applications, making them very attractive for further new nanomaterials development. The clarification of the nature of Fe-residuals in nanotubes is desirable due to their subsequent use in the synthesis of nanocomposites for theranostics materials, e.g., magnetic hyperthermia or as contrast agents for magnetic resonance.

## 2. Materials and Methods

### 2.1. Sample

The functionalized multi-walled carbon nanotubes (f-MWCNTs) with a 99% purity were purchased commercially (Smart Nanotechnologies, Cracow, Poland). The product was synthesized using a Fe-based catalyst by catalytic chemical vapour deposition (CCVD). As indicated by the producer, the pristine MWCNTs were purified in a mixture of nitric acid (HNO_3_) and sulphuric acid (H_2_SO_4_). Then, the purified nanotubes were functionalized with a carboxyl-ammonium group to obtain the final MWCNTs-COONH_4_ material subsequently denoted by us as f-MWCNTs. The detailed synthesis conditions are trade secrets. The water-soluble multiwall carbon nanotubes (MWCNTs-COONH4) exhibit an average outer diameter of about 20–25 nm (based on our TEM observations) and length from 1 to 25 µm (declared by the producer).

The carbon nanotubes were studied in pristine and calcined form. The calcination was carried out at 100 °C, 200 °C, and 300 °C under argon protection in a flow of 20 mL/min. The process was conducted in a horizontal quartz tube being a part of the oven (Czylok, Jastrzebie Zdroj, Poland) with controller temperature. The rate of temperature was 5 °C/min. The calcinated sample was stored at the final temperature for 1 h. The applied argon was with purity grade (99.999%, Linde, Cracow, Poland). The annealing temperature was chosen, taking into account future applications of f-MWCNTs as a component in spinel ferrite (SF) nanocomposites [26] to maintain the magnetic structure of SF nanoparticles. It is worth noting that initially, Fe was incorporated into MWCNTs during the synthesis process as a catalyst.

### 2.2. Methods

The crystal structure of f-MWCNTs was examined using X-ray Powder Diffraction (XRD, Malvern Instruments, Malvern, UK) using the Empyrean PANalytical diffractometer. The monochromatized Cu X-ray source of Kα_1_ = 1.54056 Å and the Bragg–Brentano geometry θ–2θ were used. All detected crystalline phases were identified based on the PDF-4 database.

For Transmission Electron Microscopy (TEM) studies, specimens were prepared by dispersing a small quantity of samples in ethanol using the ultrasonic washer (InterSonic, Olsztyn, Poland). Then, a droplet of such suspension was placed on a microscope copper grid covered with carbon. TEM studies were performed on an aberration-corrected FEI Titan electron microscope (S/TEM Titan 80-300 from FEI Co., Eindhoven, The Netherlands) operating at 300 kV.

All X-ray Photoemission Spectroscopy (XPS, Physical Electronics Inc., Eden Prairie, MN, USA) results were obtained at room temperature by using an aluminium monochromatized X-ray source of Kα = 1486.6 eV photon energy as part of the PHI 5700/660. spectrometer placed in the U.H.V. cluster. The sample was attached to the sample holder by double-coated conductive carbon tabs (PELCO Tabs™, Ted Pella Inc., Redding, CA, USA) and subsequently stored in the parking chamber under an ultra-high vacuum of 10^‒9^ Torr for about 7 days. After storage time, each studied sample was transferred into the main chamber and directly analyzed. Then, the same specimen at the same position was sputtered by Ar^+^ ion beam for 30 min, and afterwards, was subsequently measured. This ion cleaning procedure was chosen based on previous research [26]. Before analysis, each measured XPS spectra was calibrated using the C1s peak (BE = 284.8 eV) as the carbon peak usually originates from the carbon adsorbed on the surface and is used in XPS analysis as a reference for charge correction. All measured spectra were processed using MultiPak 9.6 software (Physical Electronics Inc., Eden Prairie, MN, USA). The core level lines were measured in the multiplex mode with pass energy 23.5 eV and resolution 0.1 eV. The time of measurement of each line was adjusted based on the peak intensity estimated from survey spectra. The deconvolution of the core level lines was performed by applying the Shirley-type background and the generally Gaussian–Lorentzian shape of lines. All details obtained after deconvolutions for each core level peak are provided in Supplementary Materials as separate tables. The fitting accuracy is reasonable, as expected.

The magnetic properties of the pristine nanotubes were determined using a wide-range Superconducting Quantum Interference Device (SQUID) magnetometer MPMS XL7 Quantum Design (MPMS, Quantum Design Inc., San Diego, CA, USA), while a commercial Physical Property Measurement System (PPMS) (Quantum Design Inc., San Diego, CA, USA) with Vibrating Sample Magnetometer (VSM) (Quantum Design Inc., San Diego, CA, USA) and VSM Oven options were used to study the magnetic behaviour of the annealed specimens. The thermal dependence of the DC magnetization was collected in the zero-field-cooled (ZFC) and field-cooled (FC) modes at the external field of 100 Oe and 1000 Oe, in the temperature range from 2 to 400 K, while the isothermal magnetic curves M(H) were recorded at 2 K, 100 K, and 300 K.

The Mössbauer spectra were recorded at 300 and 77 K using a standard transmission geometry equipped with a conventional constant acceleration spectrometer (Wissenschaftliche Elektronik WissEL GMBH, Munchen, Germany) and a ^57^Fe source diffused into an Rh matrix. A spectrum calibration was made with pure α-Fe powder, and the isomer shift values are quoted to α-Fe at 300K.

## 3. Results and Discussion

### 3.1. XRD

**Figure 1:**
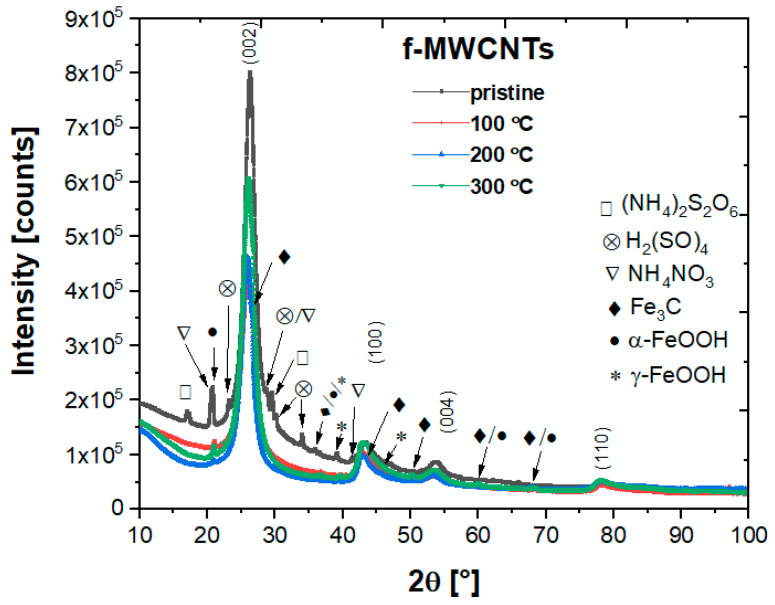
XRD diffractograms for pristine and calcined f-MWCNTs.

**Figure 2:**
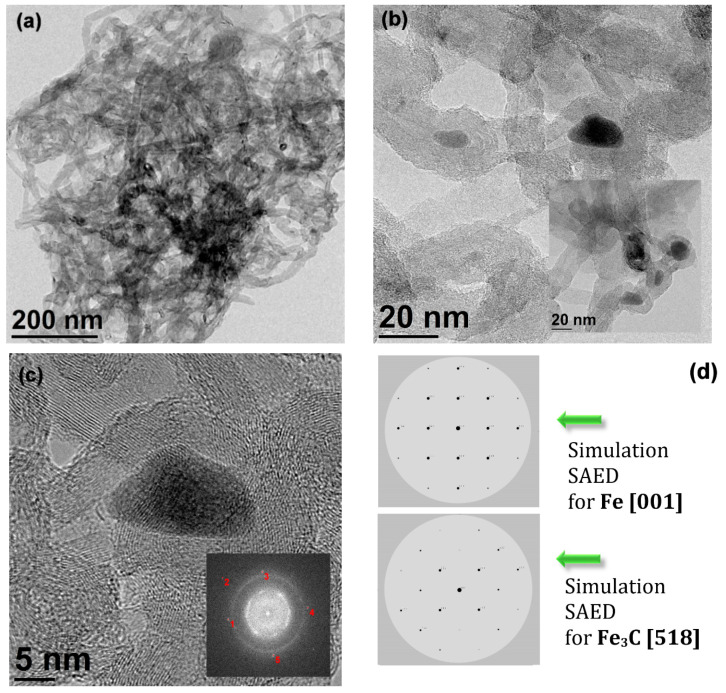
TEM image for pristine f-MWCNTs (**a**) entangled nanotubes; (**b**) nanotubes with visible Fe-based catalyst residues; (**c**) Fe-based nanoparticle with FFT analysis placed as insert; (**d**) selection area electron diffraction (SAED) pattern simulation of Fe [001] and Fe3C [518] phases.

The XRD patterns for all the f-MWCNTs specimens studied are shown in Figure 1. The XRD pattern for each studied nanotubes is complex is dominated by the presence of indexed peaks such as (002), (100), (004), and (110) at 2θ = 26.34°, 43.35°, 56.86°, 78.48°, respectively, according to ICDD reference 00-058-1638. Nevertheless, impurities as post-functionalization products are evidenced and marked by additional peaks according to the ICDD database (see Figure 1). One can notice the presence of ammonium nitrate NH_4_NO_3_ (ICDD 00-047-0864), ammonium sulfate (NH_4_)_2_S_2_O_6_ (ICDD 00-015-0241), and sulfuric acid H_2_SO_4_ (ICDD 04-014-6906). As can be seen, most of the organic impurities observed in the as-received nanotubes are removed after the calcination process. The analysis of the XRD pattern of the nanotubes is not trivial, mainly because we cannot exclude the presence of turbostratic carbon, visible as asymmetric peaks considered due to disordered graphene layers [41,42]. Indeed, TEM analysis revealed defects and damaged walls (see Figure 2). Furthermore, it has been shown that NH_3_ treatment can promote the formation of the turbostratic structure [43]. In addition, published results point out the presence of iron carbides (mainly) cementite during the synthesis of CNTs [35]. Other Fe-based contributions as catalyst residues are also discussed and studied [27,28,29,30,31,32,33,34,35,36,37,38,39,40]. However, the exact identification of each crystal phase of iron form and their content by XRD measurements is complicated due to their crystalline structures similarities.

Nevertheless, some characteristic XRD peaks are observed for both pristine and calcined nanotubes. It seems that the dominant Fe component is cementite (ICDD 00-003-0400), mainly noticeable as a bump in the nanotube peaks around θ = 26.5° and 43.6°. Some of the other Fe_3_C peaks are probably partially overlapped with those of goethite α-FeOOH (ICDD 00-001-0401) and lepidocrocite γ-FeOOH (ICDD 00-003-0079). In addition, the γ-Fe phase, which gives the most intensive peaks around 43.5° (111) and 50.5° (200) (COD crystal database card 9008469), can be overlapped with other Fe-based structures, especially that γ-Fe was detected in Mössbauer spectrometry. Thus, the precise analysis of all Fe-based structures from XRD data at this research stage is not possible the more so because of the influence of the dominated turbostratic carbon, which causes line broadening. However, the presence of all in nanotubes cannot be neglected.

### 3.2. TEM Microstructure

A representative TEM image of the f-MWCNTs is shown in Figure 2. One may notice that the bunch of functionalized carbon nanotubes is very tangled (see Figure 2a). The outer walls show many defects and are covered by amorphous layers, probably from purification products. In addition, the presence of embedded particles filling the inner cavity of the nanotube and varying in size is evident (see Figure 2b). Thus, these nanoparticles have an average size of 11–22 nm and can be recognized as post-synthesis catalyst residues.

The inset in Figure 2c shows the diffractogram taken on the particle displayed in the image. By analyzing the FFT, we can determine the interplanar spacing, and with well-defined graphite spacings, we can use the standard to identify Bragg reflections. Thus, we can deduce that the observed diffraction can be recognized as Fe (*Fm-3m*) in the direction of [001] or Fe_3_C diffraction in the direction of [518]. The latter corresponds better to the visible diffraction spots. Thus, for example, reflex numbers 1 and 3 observed with an interplanar spacing d of 1.82 Å can be recognized as Fe(200) (tabular value—1.71 Å) or Fe_3_C (13-1) (tabular value—1.87 Å), which is a better match to Fe_3_C. Thus, the two diffractions are similar, the angles are the same, and the spacings are very close, and it is not easy to decide which structure is visible in the embedded particles, which can be well demonstrated by the simulated SAED for Fe [001] and Fe_3_C [518] (see Figure 2d). Probably, we have a co-existing fraction of Fe_3_C, Fe, and possibly even iron oxides [32,44,45,46]. The applied calcination procedure does not drastically change the microstructure of the f-MWCNTs. It is worth adding that the cementite structure is composed of hexagonal close-packed layers of Fe atoms at two different sites with slightly different magnetic moments while carbon atoms occupy the interstitial sites.

### 3.3. Raman Spectroscopy

**Figure 3:**
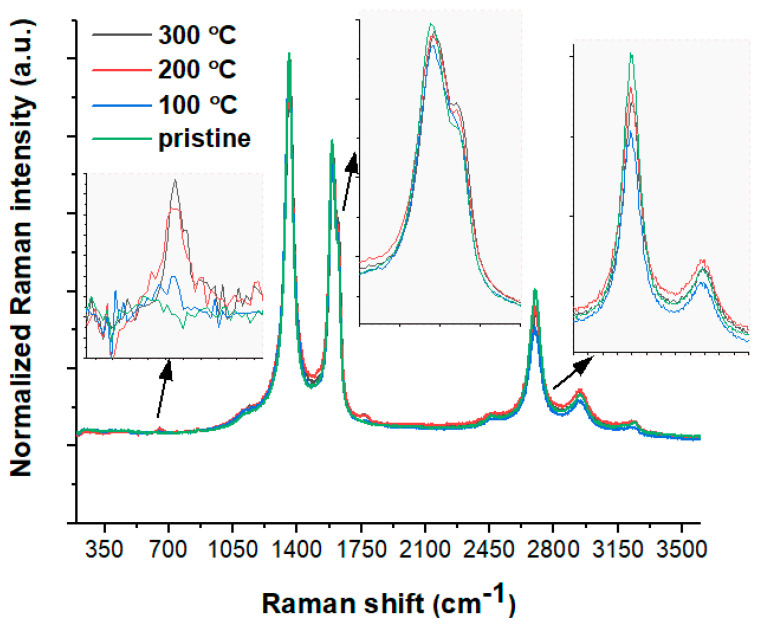
Raman spectra of pristine and calcined f-MWCNTs presented in the 200–3600 cm^−1^ range.

The Raman spectra of the pristine and heat-treated carbon nanotubes are summarized in Figure 3, while the Raman band positions and the intensity ratio are presented in Table 1. It is interesting to note that the spectrum of pristine CNTs displays D and G bands located at around 1360–1590 cm^−1^, which are associated, respectively, with defective and disordered graphitic layers on the surface wall [45,47,48] as well as with in-plane vibrations of the graphite sheet [48]. In this context, the D band is generally attributable to disordered amorphous carbon but not the defects inside tube walls [49], while the G band to the (C=C) stretching vibration within the carbon ring. According to the literature, the D band is generally assigned to the A1g symmetry mode [47], while the G band corresponds to the E2g modes [50,51]. The strong D and G modes indicate a defective structure of the studied carbon nanotubes but without significant sidewall damage. Pristine carbon nanotubes are also loaded with tube surface defects due to a clearly visible D′ band around 1620 cm^–1^ [52].

The Raman spectra of the studied CNTs also revealed the D* band at about ~2690 cm^−1^, which is the second harmonic of the D band (see Table 1) [47,53]. According to the literature, the intensity of the D* band corresponds to the extent of impurities in the sample [49] or results from the mass fraction, i.e., the decrease in D* intensity translates into a decrease in the CNT mass [53]. A very similar arrangement of Raman bands was found for the heat-treated samples with only slight differences in intensity and band positions (see Table 1).

Furthermore, two interesting observations were made when comparing the heat-treated samples with the pristine material. First, a tiny Raman band around 700 cm^–1^ appeared on the CNTs sintered at 200 °C and 300 °C, suggesting a surface evolution of the carbon nanotubes or, alternatively, point to the precipitation of some impurities or metals. Secondly, the decrease in the D* value as a function of the annealing temperature of the nanotubes may suggest a decrease in the CNT mass fraction (Table 1). The data mentioned before can be used to probe the quality of carbon materials. Here, the ratio between the intensities of the D and G bands or D and D* is generally useful for the characterization of carbon materials [54]. The changes in the I_D_/I_G_ and I_D_/I_D_* ratios for each sample are given in Table 1. Generally, the low value of I_D_/I_G_ ≈ 0.23 suggests a good quality of the carbon material. In the case of the pristine nanotube, the I_D_/I_G_ ratio is higher than 1.50, indicating a higher amount of amorphous carbon. What is more interesting is that the I_D_/I_G_ values for the heat-treated samples are generally close to each other but are much higher than the value for the pristine material. Such behaviour could suggest the local increase in disordered amorphous carbon. The Raman study also allows the purity of the carbon materials to be checked by calculating the I_D_/I_D_* ratios. The higher the I_D_/I_D_* value, the purer the material studied. Our studies showed very high I_D_/I_D_* values for all samples, suggesting relatively low carbon impurities, such as aliphatic moieties or methyl groups.

### 3.4. X-ray Photoemission Spectroscopy (XPS)

**Figure 4:**
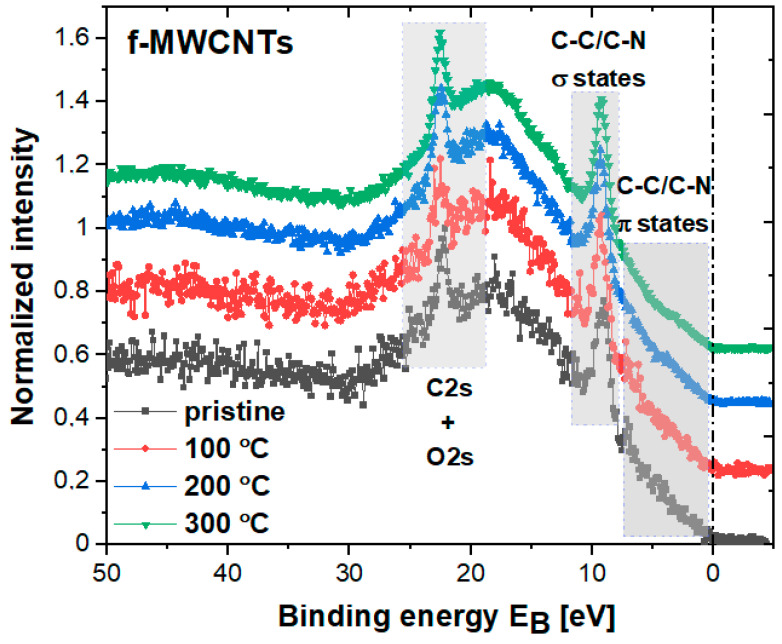
The comparison between valence bands in pristine and calcined f-MWCNTs samples.

The electronic structure of various carbon nanotubes can be effectively studied by XPS measurements [17,18,19,20,21,55,56,57,58,59]. However, it is important to remember first that the sensitivity of XPS is limited to the surface with a depth of about 10 nm for the monochromatized Al source. The changes in the valence band (VB) spectra as a function of sample treatment are illustrated in Figure 4. There is no significant difference in VB between all samples, meaning that the calcination process did not affect the high degree of crystallinity of the CNTs wall structure. However, after annealing at 300 °C, a slight increase in the states around the Fermi level (E_F_) can be noted, which can probably be associated with the influence of the Fe-based magnetic components modified over heat treatment. Nevertheless, such states with a noticeable bump around 4.1 eV may also be evidence of sp^2^ hybridized nitrogen as photoelectrons emitted from nitrogen lone pair states, as already demonstrated by Ruelle et al. [17].

By analyzing the VB, one can notice a distinguishable peak in the binding energy range of about BE ≈ 7.4–11 eV with the maximum at about BE ≈ 9.3 eV that be assigned to overlapped C–C/C–N 2p-σ states, probably also enhanced by C–O states [60]. Similarly, between E_F_ and 7 eV, the relatively flat states can be attributed to C–C/C–N 2p-π states. The next prominent peak between 20 eV and 24 eV may originate from overlapped C2s and O2s states. The valence band of carbon nanotubes has been successfully described by, e.g., Ruelle et al. [17] and Nesov et al. [59]. The band structure pattern determined is similar to that observed by us, but the sharp and prominent peaks in the VB noted in our studies and described as C–C/C–N 2p–σ states and C/O 2s states are a rather new phenomenon. So far, these states have been described as broad bands. Here, the first peak can probably be associated with various N-based groups, e.g., graphitic nitrogen, where a nitrogen atom is substituting a carbon atom and is incorporated into the graphene layer. It appears that the VB configuration is strictly associated with the way of CNTs synthesis and further functionalization.

**Figure 5:**
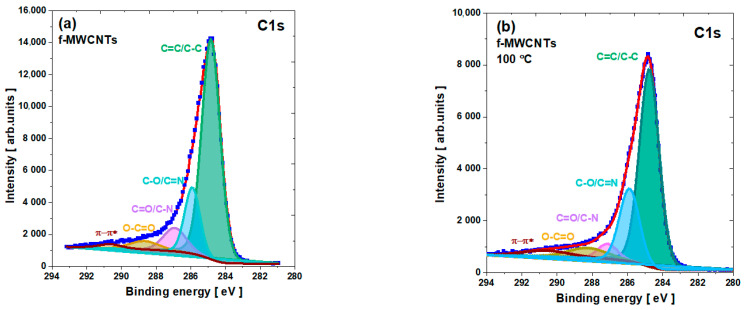

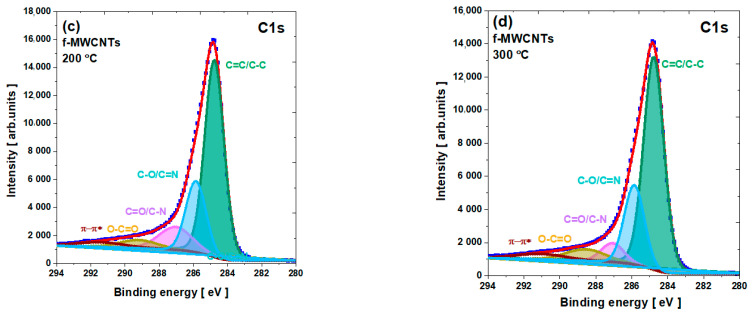
The C1s core level line for f-MWCNTs: (**a**) pristine; calcined at (**b**) 100 °C; (**c**) 200 °C; and (**d**) 300 °C.

The C1s core level lines (see Figure 5) are asymmetric and can be deconvoluted into five components as for carbon nanotubes [17,18,19,20,21,55,56,57,58,59] (see Table S1 in Supplementary Materials). The first one, around 284.8 eV, which is dominant, can be assigned to the C=C sp^2^ carbon bonds, which are rather overlapped with the sp3 C–C bonds. It is interesting to note that Fe–C states from catalyst residues can also overlap this peak, but their content is relatively low. These states are usually visible as small peaks around 283.5 eV, indicating a rather strong interaction between Fe3C and the surface of the graphene layer [61,62]. The next peak, around 285.9 eV, represents the C–O and C=N state, while the one around 287 eV may represent C=O overlapped with sp3 C–N. The following broad line, above 288.6 eV, can be attributed to the carboxyl O–C=O component. The last broad line with the lowest intensity near 290 eV can be identified as a satellite π-π-* shake-up from the sp^2^ hybridized carbon.

**Figure 6:**
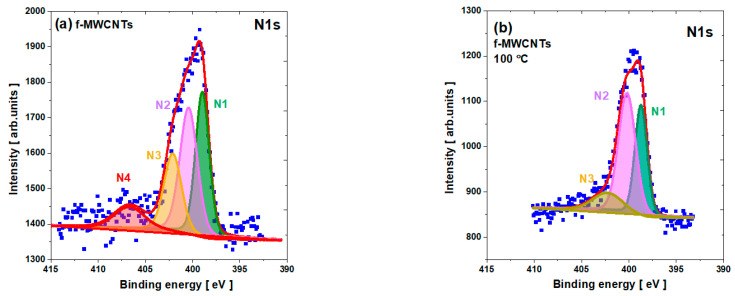

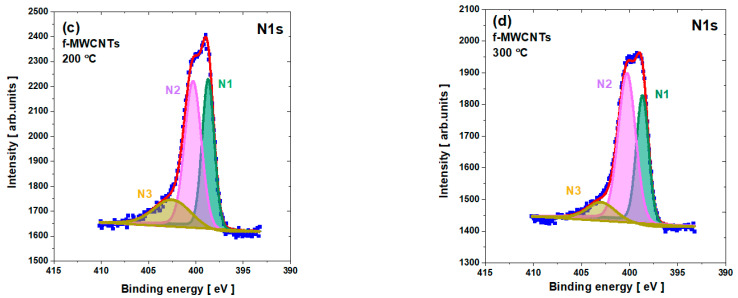
The N1*s* core level line for f-MWCNTs: (**a**) pristine; (**b**) calcined at 100 °C; (**c**) calcined at 200 °C; and (**d**) calcined at 300 °C.

The N1s spectra of pristine and calcined nanotubes reveal several contributions related to N-based functionalities (see Figure 6), namely N1, N2, N3, and N4 (see Table S2 in Supplementary Materials). The N1 peaking around 399 eV can be identified as pyridine and nitryl groups; N2 around 400 eV to amide, amine, lactam, and pyrrole; the N3 observed around 402 eV to quaternary nitrogen atoms bonded to three C atoms in the bulk of the graphene layers; the broad N4 peak around 406 eV to pyridine-N-oxide overlapped with probably dominated adsorbed N^2^ [4,18,21,26]. The relative percentage of the different N-species was determined for each sample based on the deconvoluted spectra. For the pristine f-MWCNTs, we observe all four nitrogen-based lines, whereas, for the calcined specimens, only three components are visible with different relative percentage contributions. Moreover, the N2 component dominates for both pristine and calcined nanotubes. This contribution is probably enhanced with Fe–N states due to the presence of Fe/Fe_3_C catalyst residues [62]. N3 is reduced after calcination to about 9 at. % for the sample calcined at 300 °C. The modification of the nitrogen-based group during the calcination process has already been observed, for example, by Kundu et al. [29], who studied CNTs heat-treated under NH_3_ at different temperatures. As in the case of our samples, he determined the domination of the N2 states for the sample annealed at 200 °C.

**Figure 7:**
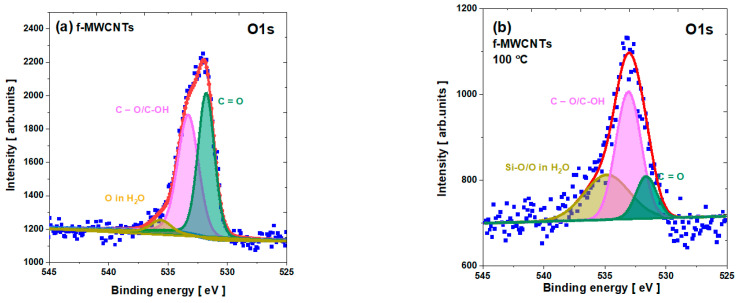

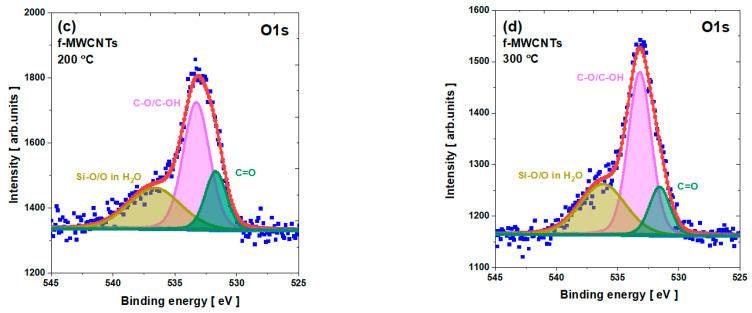
The O1s core level line for f-MWCNTs: (**a**) pristine; (**b**) calcined at 100 °C; (**c**) calcined at 200 °C; and (**d**) calcined at 300 °C.

The O1*s* line is complex for all the nanotubes studied and can be attributed to three species (see Figure 7) (see Table S3 in Supplementary Materials). The complexity of the O1*s* line can be derived from various components related to C=O (BE ≈ 531.6 eV), C–O/C–OH (BE ≈ 533.2 eV), and the peak at about 536 eV. The first peak dominates pristine nanotubes, while the second is dominant for calcined nanotubes. Moreover, the last peak for the pristine f-MWCNTs can probably be caused by physisorbed water as an effect of functionalization in the wet environment, while for the calcined samples, this peak becomes very broad, probably due to the overlap with the Si–O states as an effect of the nanotube annealing on the quartz boat. It should also be emphasized that the second peak can be enhanced by the N–O bonding sites generally noticeable around 534 eV.

**Figure 8:**
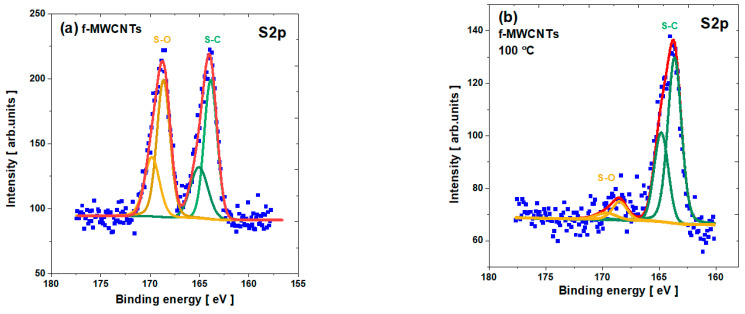

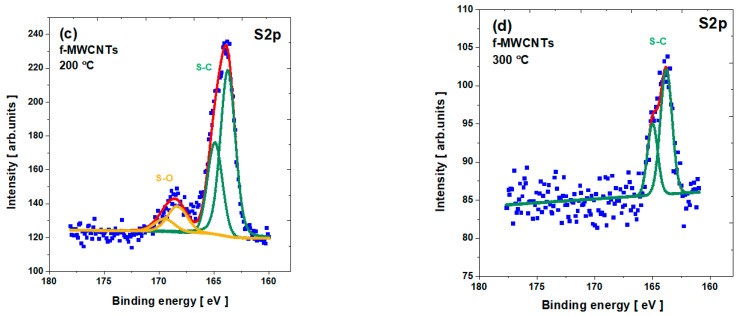
The S2p core level line for f-MWCNTs: (**a**) pristine; (**b**) calcined at 100 °C; (**c**) calcined at 200 °C; and (**d**) calcined at 300 °C.

The analysis of the S2p spectra indicates a small contribution of the sulfur-based components (see Figure 8) as residues from the purification of the nanotubes (see Table S4 in Supplementary Materials). Nevertheless, the sulfur line exhibits two prominent peaks that can be recognized as C–S–C and S–O states. The first is visible as two distinct peaks S2p_3/2_ and S2p_1/2_, around BE ≈ 163.7 eV and BE ≈ 164.9 eV, respectively, and probably enhanced by the Fe-S states [62]. The second states are also revealed by two peaks, S2p_3/2_ and S2p_1/2_, around BE ≈ 168.5 eV and BE ≈ 169.7 eV. In the case of pristine nanotubes, both sulfur states are noted. However, as can be observed, the calcination procedure slightly reduces the S–O states until they disappear entirely after annealing at 300 °C. Concomitantly, sulfur-carbon states as a covalent bond are visible for all the studied samples.

**Figure 9:**
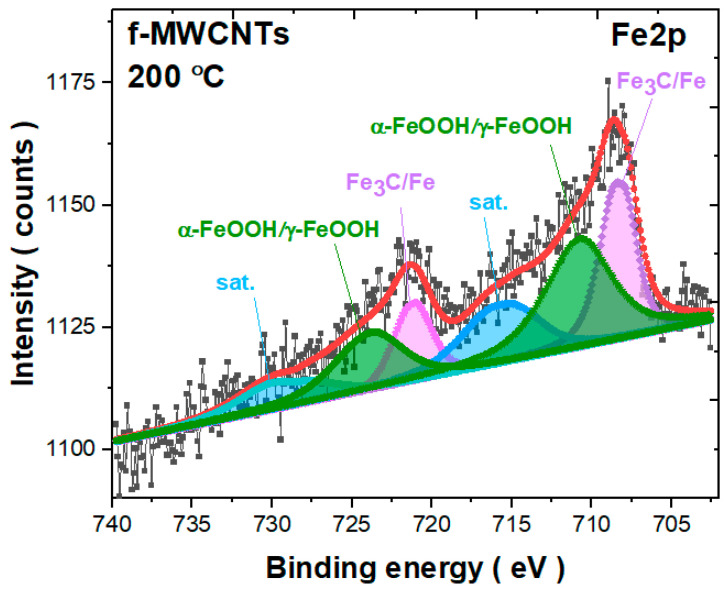
The Fe2p core level line for f-MWCNTs annealed at 200 °C.

The example of the Fe2p line measured for f-MWCNTs calcined at 200 °C is shown in Figure 9 (see Table S5 in Supplementary Materials). The deconvolution was performed based on the Doniach–Sunjic (DS) line shape [63]. Obviously, the iron line is rather weak due to the low iron content of the sample. The peaks around 708 eV and 721 eV can be assigned to overlapped states of Fe_3_C (cementite) and Fe with a dominant contribution of iron carbide [61,62,64]. The next two broad peaks around 710.6 eV and 723.8 eV are rather ascribed to γ–FeOOH (lepidocrocite) or/and α–FeOOH (goethite) states [65,66]. According to previous studies, the binding energy difference between the two components is only 0.1 eV [65,66]. Considering the sensitivity of our spectrometer, which is about 0.3 eV, we are not able to precisely distinguish which component dominates in the studied material. Instead, we can deduce that it could be a convolution of the two species due to the broad peaks. There are also two additional satellite peaks. The relative contribution of each state estimated based on the deconvolution is about 31.3 at.%, 44.5 at.%, and 24.3 at.%, respectively. Thus, it appears that based on the fit of the Fe2p photoemission line, the overlapping goethite/lepidocrocite states dominate that of cementite. However, one should be aware that a broad satellite structure may be composed of states assigned to both Fe-based contributions, and their ratio can be disturbed.

### 3.5. Magnetic Properties

**Figure 10:**
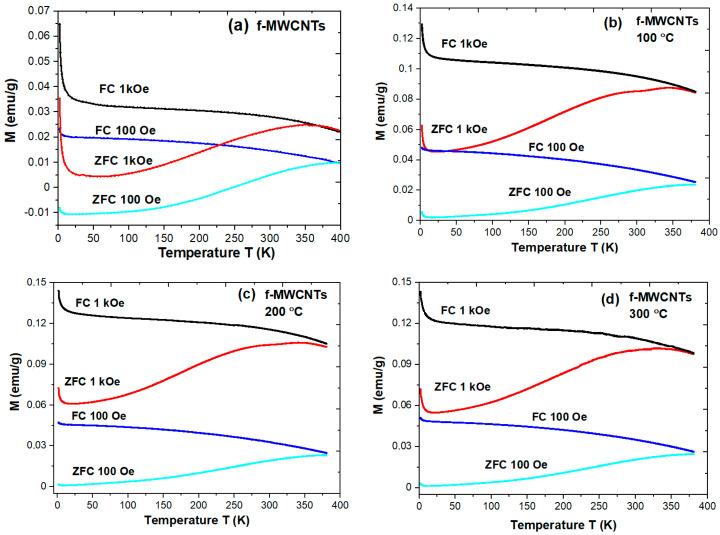
The thermomagnetic dependence of magnetization M(T) measured at 1 kOe and 100 Oe for f-MWCNTs: (**a**) pristine; (**b**) calcined at 100 °C; (**c**) calcined at 200 °C; (**d**) calcined at 300 °C.

The thermal evolution of the magnetization measured under zero-field-cooled (ZFC) and field-cooled (FC) conditions under an applied field of 1 kOe and 100 Oe is depicted in Figure 10. As one may notice, the magnetization is relatively lower for pristine nanotubes than for calcined ones. In addition, for calcined nanotubes, the modification of thermal dependence of magnetization seems to be independent of the calcination temperature. However, it is worth noting that the estimated difference M_FC_-M_ZFC_ is slightly dependent on calcination procedure from about 0.029 emu/g (pristine) to 0.048 emu/g (calcined at 300 °C) and 0.031 emu/g (pristine) to 0.070 emu/g (calcined at 300 °C) estimated at 100 Oe and 1 kOe, respectively. Thus, the observed differences can probably be related to variation within Fe-based components modified over calcination. The difference in the ZFC and FC curves and their divergence above room temperature revealed some thermal blocking effects, usually attributed to magnetic anisotropy. Indeed, the maximum occurrence in ZFC curves can be associated with the blocking process of some Fe-complexes as nanoparticle catalyst residues. The observed M(T) variations are relatively consistent with previously published results [23,34]. Jamrozik et al. [34] revealed that the M(T) dependence at a lower temperature for all studied nanotubes is different than expected for pure cementite, suggesting the influence of additional iron-based species, which lead to a slight increase in magnetization with decreasing temperature. As the authors claim, the magnetization results demonstrate a predominant amount of iron in the ferromagnetically ordered (or in the form of superparamagnetic particles) cementite phase. Smaller magnetization values for MWCNTs samples than that of bulk Fe_3_C can also be caused by some additional diamagnetic elements such as oxygen present in the samples [23,34]. Indeed, as shown by XRD and XPS analysis, the diamagnetic elements are observed in all studied samples, but their relative contribution is reduced after calcination. Probably, therefore the magnetization is lower for pristine nanotubes than for annealed ones (see Figure 10). In addition, we also observe an evident increase in magnetization at the low-temperature range (below 20 K for 1 kOe and below 12 K for 100 Oe), which points to the conclusion that probably in our samples, there are more than one Fe-based species.

**Figure 11:**
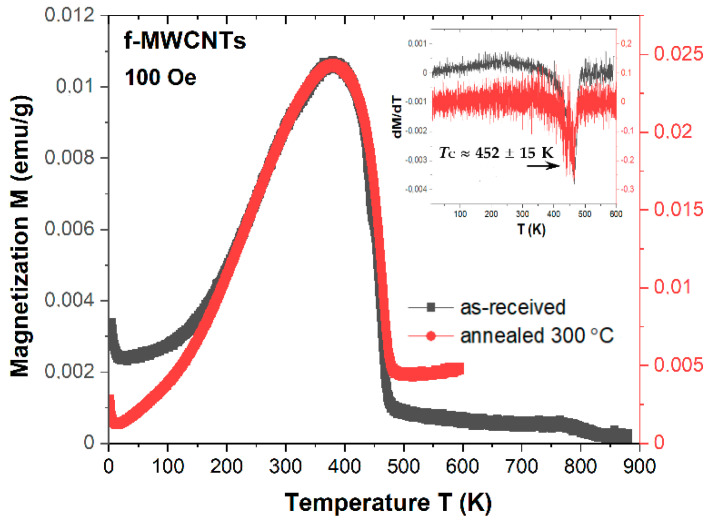
The ZFC dependence of magnetization M(T) measured at 100 Oe for f-MWCNTs and calcined at 300 °C. Inset shows the dM/dT vs. T dependence.

The M_ZFC_(T) high-temperature measurements performed for pristine and calcined at 300 °C nanotubes allow determining the phase transition temperature for magnetic component dominated in f-MWCNTs. Between 400–500 K, the evident phase transition can be noted. The performed dM/dT differentiation allows estimating the value of the Curie temperature, which equals T_C_ ≈ 452 ± 15 K. As previously discussed, in the studies samples, we can distinguish several types of Fe-based structures with the evident influence of Fe_3_C. Cementite is a metallic ferromagnet transforming to the paramagnetic state at a Curie temperature of about T_C_ = 483 K [67]. In our case, such temperature is lower, but as evidenced, the presence of other magnetic Fe-based components cannot be neglected. Indeed, Lee et al. [68] revealed the magnetic phase transition for goethite (α-FeOOH) and lepidocrocite (γ-FeOOH) nanoparticles. Both crystallize in the orthorhombic structure and show the antiferromagnetic arrangement with the Néel temperature T_N_ = 252 K and 53 K, respectively. Such values are lower than bulk materials, e.g., for bulk α-FeOOH, it equals about T_N_ = 372 K [69]. Thus, the origin of the broad peak observed for f-MWCNTs may comprise the overlapped magnetic phase transition of dominated cementite and partly goethite. The influence of lepidocrocite is low and can be noted by a weak inflexion point around 50 K. However, there is an additional peculiarity between 750 K and 850 K as the induced heat-treatment phase transition during heating the sample under measurements conditions above 300 K. As evidenced by Boi et al. [70], the decomposition of γ-Fe into α-Fe starts even at temperatures below 200 °C and is significantly faster in the temperature range of 300–399 °C. However, such a phenomenon occurs in a strictly controlled environment without possible oxidation. The magnetic transition presented in Figure 11 at high temperatures is lower than for typical α-Fe, which may indicate that the Fe-oxides are probably generated during measurements.

**Figure 12:**
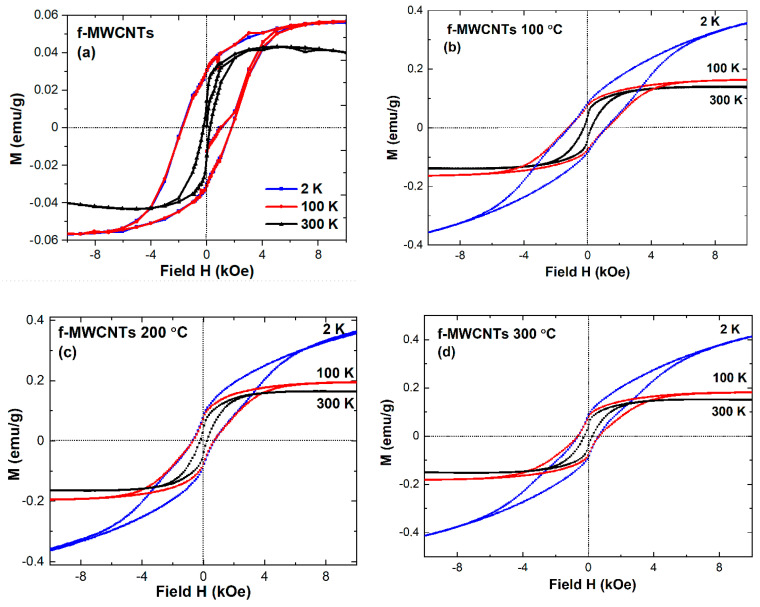
Hysteresis loops *M*(*H*) measured at 2 K, 100 K, and 300 K for f-MWCNTs: (**a**) pristine; (**b**) calcined at 100 °C; (**c**) calcined at 200 °C; (**d**) calcined at 300 °C.

The analysis of hysteresis loops was conducted at 2 K, 100 K, and 300 K (see Figure 12). Generally, all studied samples revealed ferromagnetic-like behaviour with evident coercivity (H_C_) and some diamagnetic contribution. The first one is evidence of Fe-based nano-components, while the latter may originate from non-magnetic carbon and functional groups component as well as the possible influence of the sample holder. In order to estimate saturation magnetization just dependent on Fe species present in the sample, the diamagnetic component can be separated from the ferromagnetic one. Such separation can be achieved by subtracting the diamagnetic part by linear fitting of M(H) at a higher field. Using such an approach, we can distinguish the magnetization values at the highest applied external field (placed in Table 2 as M_7T_ for pristine and M_9T_ for calcined nanotubes) and the values of saturation magnetization M_S_ of the ferromagnetic component as the extrapolation, which is obviously different. Thus, the analysis of saturation magnetization values shows a significant difference between pristine and calcined samples, where M_.S._ differs almost by order of magnitude. Nevertheless, for all calcined samples, such value is almost the same (see Table 2). The shape of hysteresis loops reveals a small deformation at 300 K for pristine and 100 °C for calcined nanotubes and even more noticeable at 200 °C and 300 °C for calcined nanotubes at just 2 K. The observed deformation can be associated with various Fe-based components and their exchange coupling behaviour.

Concomitantly, the magnetization process is sensitive to the synthesis type and purification and functionalization process of nanotubes. As previously published, e.g., by Lipert et al. [71], the magnetic properties of CNTs prepared with magnetic catalyst material are determined by the ferromagnetic or superparamagnetic catalyst particles formed during synthesis and remaining inside carbon nanotubes after such process. As reported during the synthesis of CNTs, some catalyst particles are encapsulated into nanotubes forming a single magnetic domain and, in this way, enhancing the coercivity of nanotubes. Such phenomena were observed in pure and functionalized nanotubes and their composites [5,26]. The value of coercivity estimated based on hysteresis loops is reduced upon calcination from 1.77 kOe (pristine f-MWCNTs) to 0.63 kOe (calcined at 300 °C f-MWCNTs). Such a reduction is also well seen at 100 K, but at room temperature, coercivity seems to be relatively stable (see Table 2). The observed modification of coercivity is probably related to various Fe-based species. Generally, for nanocrystalline Fe_3_C with a particle size of about 22 ± 4 nm, the H_C_ estimated at 300 K is about 0.173 kOe [72]. It is worth noting that for nanocrystalline Fe_3_C, the magnetization is lower than for bulk specimens due to the smaller value of particles size [72].

In our case, for pristine f-MWCNTs at 300 K, we estimated the H_C_ value as about 0.238 kOe, which is slightly higher than the value observed for pure cementite. As further evidence, we have more than one iron type in the studied nanotubes. So far, the value of coercivity for nanotubes was also estimated by Jamrozik et al. [34], who showed that for MWCNTs-COOH, the coercivity is varied from about 2 kOe (3 K) to 0.28 kOe (295 K). The latter studies performed by Jamrozik et al. [40] for control and as-milled MWCNTs-COONH_4_ nanotubes demonstrate the value of H_C_ as about 1.24 kOe (3 K) and 0.27 kOe (295 K) for as-prepared specimens. Thus, the values noted by us (see Table 2) are in reasonably good agreement with those recently published. The authors revealed that milling has a slight influence on coercivity. However, the variation of magnetization compared in as-prepared and milled samples can be affected by changes within iron phases, their individual content, and chemical composition. The contribution of various iron species was determined by the Mössbauer spectrometry.

### 3.6. Mössbauer Spectrometry

**Figure 13:**
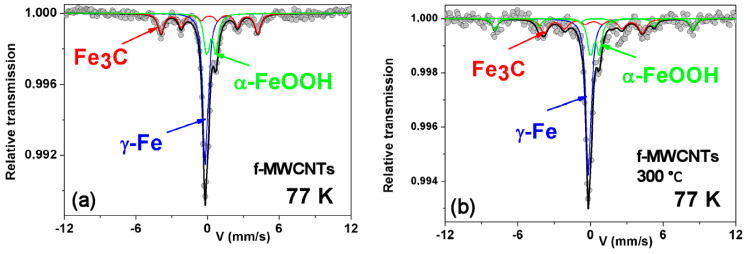
Mössbauer spectra measured at 77 K for f-MWCNTs: (**a**) as-received; (**b**) calcined at 300 °C.

The main objective of using ^57^Fe Mössbauer spectrometry is to determine the form of iron in the studied nanotubes, but the first difficulty has related to the preparation of the Mössbauer sample due to the low content of Fe. The measurements carried out with a very intense radioactive source (about 1.6 GBq) only at 77 K, required about two weeks registration time to obtain good statistics, as shown in Figure 13 with the relative transmission scale. The hyperfine structure of the pristine f-MWCNTs consists of a single predominant line, a magnetic sextet, and a quadrupolar doublet. The refined values of the hyperfine parameters of these three components are listed in Table 3 and allow us to assign them unambiguously to γ-Fe, cementite, and some Fe^3+^ oxide phase, respectively. After annealing at 300 °C, the main difference is due to the presence of an additional magnetic sextet attributed to a Fe^3+^ oxide phase. A rigorous analysis leads to attributing the quadrupolar doublet and the second magnetic sextet to goethite nanoparticles. Nevertheless, at this stage, in agreement with previous XPS results, the presence of some traces of lepidocrocite (γ-FeOOH), which exhibit rather similar values of hyperfine parameters in the paramagnetic state, cannot be excluded. Indeed, it can be concluded that the conditions of synthesis favor the emergence of some γ-Fe (nano)particles probably embedded in a goethite shell after oxidation, in addition to cementite grains and goethite nanoparticles (probably smaller than about 10 nm). In contrast, annealing at 300 °C should favour the aggregation of the goethite nanoparticles, giving rise to larger magnetically blocked nanoparticles of goethite. However, it is essential to note some disagreements in the estimated proportions in the pristine and annealed samples: they can be attributed not only to the differences in the Lamb–Mössbauer factors of the three components, but it must also be taken into account that the errors are amplified due to the very low content of Fe. However, it is very important to note that the present results agree quantitatively with those presented earlier [34,35,36,37,38,39,40].

## 4. Conclusions

This study presents the complete study of commercial f-MWCNTs functionalized by the -COONH_4_ group. We analyzed both pristine and calcined nanotubes using different and complementary techniques. As demonstrated, the Fe-base embedded nanoparticles as synthesis residues are responsible for the magnetic behaviour. Based on XRD and ^57^Fe Mössbauer spectrometry, we have identified three main Fe-based species: γ-Fe, cementite (Fe_3_C), goethite (α-FeOOH). Diffraction analysis indicates an additional phase of lepidocrocite (γ-FeOOH), which is also not excluded in the photoemission and Mössbauer studies. However, the amount of each constituent is low. The average size of NPs inside nanotubes is between 11–22 nm. The Fe-based magnetic nanoparticles of cementite are blocked above room temperature, as shown by ZFC curves. The magnetic properties are slightly modified during the heat treatment due to the modification within iron components. From the thermomagnetic curves, the typical phase transition of cementite is observed. However, Mössbauer spectrometry revealed the domination of γ-Fe over Fe_3_C and α-FeOOH. Diffraction, Raman, and microscopic analysis show that the carbon graphene layers in the nanotubes are disordered due to the turbostratic form of the carbon. The photoemission studies showed almost stable states of the carbon but a significant modification in the nitrogen and oxygen groups as an effect of calcination. Indeed, the reduction in organic impurities during calcination is evident from the XRD pattern.

The performed multi-technique characterization of f-MWCNTs nanotubes provided sufficient information on their structural, magnetic, and spectroscopic behaviour. This analysis could be helpful for the design and development of nanocomposites based on nanotubes as functional materials with a wide range of applications.

## Figures and Tables

**Table 1:** Gaussian and Lorentzian fitting results of Raman spectra taking into account bands D, G, D′, and D*, the intensity ratio of I_D_/I_G_, I_D_/I_D_*, and FWHM of pristine and calcinated carbon nanotubes.

f-MWCNTs	D Band Position (cm^–1^)	G Band Position (cm^–1^)	D’ Band Position (cm^–1^)	D* Band Position (cm^–1^)	I_D_/I_G_	I_D_/I_D_*
**pristine**	1357.4	1587.1	1619.6	2693.1	1.89	2.45
**100 °C**	1357.8	1587.6	1619.5	2691.8	2.14	3.51
**200 °C**	1358.3	1587.5	1619.6	2692.8	2.12	3.26
**300 °C**	1359.1	1588.6	1620.1	2694.9	1.98	2.89

**Table 2:** Magnetic properties of pristine and calcined f-MWCNTs.

Samples	2 K				100 K				300 K			
**f-MWCNTs** **pristine**	**M_7T_** **(emu/g)** **±0.006**	**M_S_** **(emu/g)** **±0.006**	**M_R_** **(emu/g)** **±0.006**	**Hc** **(kOe)** **±0.005**	**M_7T_** **(emu/g)** **±0.006**	**M_S_** **(emu/g)** **±0.006**	**M_R_** **(emu/g)** **±0.006**	**Hc** **(kOe)** **±0.005**	**M_7T_** **(emu/g)** **±0.006**	**M_S_** **(emu/g)** **±0.006**	**M_R_** **(emu/g)** **±0.006**	**Hc** **(kOe)** **±0.005**
	0.02	0.07	0.03	1.77	0.02	0.07	0.031	1.78	−0.04	0.05	0.01	0.24
**f-MWCNTs** **calcined**	**M_9T_** **(emu/g)** **±0.006**	**M_S_** **(emu/g)** **±0.006**	**M_R_** **(emu/g)** **±0.006**	**Hc** **(kOe)** **±0.02**	**M_9T_** **(emu/g)** **±0.006**	**M_S_** **(emu/g)** **±0.006**	**M_R_** **(emu/g)** **±0.006**	**Hc** **(kOe)** **±0.02**	**M_9T_** **(emu/g)** **±0.006**	**M_S_** **(emu/g)** **±0.006**	**M_R_** **(emu/g)** **±0.006**	**Hc** **(kOe)** **±0.02**
**100 °C**	0.40	0.53	0.08	1.18	0.07	0.19	0.08	1.23	0.01	0.16	0.39	0.26
**200 °C**	0.54	0.47	0.08	0.74	0.11	0.22	0.08	0.69	0.01	0.19	0.40	0.22
**300 °C**	0.51	0.63	0.09	0.63	0.05	0.21	0.08	0.65	−0.03	0.18	0.44	0.24

**Table 3:** Refined values of hyperfine Mössbauer parameters in pristine and calcined f-MWCNTs determined at 77 K: δ: isomer shift; Γ: full width at half height; Δ: quadrupolar splitting; 2ε: quadrupolar shift; B_hf_: hyperfine field.

Samples	77 K					
**f-MWCNTs** **pristine**	**δ** **(mm/s)** **±0.02**	**Γ** **(mm/s)** **±0.02**	**Δ or 2** **ε** **(mm/s)** **±0.02**	**B_hf_** **(T)** **±1.0**	**(%)** **±3**	**Component**
	−0.01	0.49	0 *	–	49	γ-Fe
	0.43	0.68	0 *	24.9	32	Fe_3_C
	0.37	0.33	0.90	–	19	α-FeOOH
**f-MWCNTs** **calcined at** **300 °C**	**δ** **(mm/s)** **±0.02**	**Γ** **(mm/s)** **±0.02**	**Δ or 2** **ε** **(mm/s)** **±0.02**	**B_hf_** **(T)** **±1.0**	**(%)** **±3**	**component**
	0.02	0.30	0 *	–	41	γ-Fe
	0.44	0.77	0.11	25.7	26	Fe_3_C
	0.46	0.74	0.70	–	20	α-FeOOH
	0.50	0.50	−0.22	50.5	13	α-FeOOH

## Data Availability

The data presented in this study are available on request from the corresponding author. The data are not publicly available due to restrictions of privacy.

## Supplementary Materials

The following are available online at https://www.mdpi.com/article/10.3390/ma15030977/s1; Table S1: The results of C1s core level lines fit pristine and calcined f-MWCNTs nanotubes; Table S2. The results of O1s core level lines fit pristine and calcined f-MWCNTs nanotubes; Table S3. The results of N1s core level lines fit pristine and calcined f-MWCNTs nanotubes; Table S4. The results of S2p core level lines fit pristine and calcined f-MWCNTs nanotubes; Table S5. The results of Fe2p core level lines fit for f-MWCNTs nanotubes calcined at 200 °C.
