# Supplementary material for: A Comprehensive Study of Pristine and Calcined f-MWCNTs Functionalized by Nitrogen-Containing Functional Groups
Source: Materials (Basel). 2022 Jan 27;15(3):977. doi: 10.3390/ma15030977 (PMC8838665; doi:10.3390/ma15030977)
Supplement: Supplementary file 1 [file materials-15-00977-s001.zip › materials-1547156-supplementary.pdf]

Supplementary Materials

A Comprehensive Study of Pristine and Calcined f-MWCNTs Functionalized by Nitrogen-Containing Functional Groups

Anna Bajorek 1,2,*, Bogumiła Szostak 1,2, Mateusz Dulski 2,3, Jean-Marc Greneche 4, Sabina Lewińska 5, Barbara Liszka 6, Mirosława Pawlyta 7 and Anna Ślawska-Waniewska 5

1 A. Chełkowski Institute of Physics, University of Silesia in Katowice, 75 Pułku Piechoty 1, 41-500 Chorzów, Poland; szostakbogumila@gmail.com
2 Silesian Center for Education and Interdisciplinary Research, University of Silesia in Katowice, 75 Pułku Piechoty 1A, 41-500 Chorzów, Poland; mateusz.dulski@us.edu.pl
3 Institute of Materials Science, University of Silesia in Katowice, 75 Pułku Piechoty 1A, 41-500 Chorzów, Poland
4 Institut des Molécules et Matériaux du Mans UMR CNRS 6283, Le Mans Université, Avenue Olivier Messiaen, CEDEX 9, 72085 Le Mans, France; jean-marc.greneche@univ-lemans.fr
5 Institute of Physics, Polish Academy of Sciences, Al. Lotników 32/46, 02-668, Warsaw, Poland; lewinska@ifpan.edu.pl (S.L.); slaws@ifpan.edu.pl (A.Ś.-W.)
6 Faculty of Natural Sciences, University of Silesia in Katowice, Będzińska 60, 41-200 Sosnowiec, Poland; barbara.liszka@us.edu.pl
7 Materials Research Laboratory, Institute of Engineering Materials and Biomaterials, Silesian University of Technology, Konarskiego 18A, 44-100 Gliwice, Poland; miroslawa.pawlyta@polsl.pl
* Correspondence: anna.bajorek@us.edu.pl

Table S1. : The results of C1s core level lines fit pristine and calcined f-MWCNTs nanotubes. .

Sample	Line	Component	Peak position [eV]	Area [%]	FWHM	χ–Squared
f-MWCNTs	C1s	C–C/C=C	284.83	66.90	1.25	4.78
		C–O/C=N	285.91	16.90	1.05	
		C=O/C–N	286.93	9.48	1.56	
		O–C=O	288.68	4.87	2.06	
		π-π*	290.64	1.85	1.62	
f-MWCNTs 100°C	**C1s**	C–C/C=C	284.78	62.05	1.33	2.33
		C–O/C=N	285.89	21.90	1.33	
		C=O/C–N	287.14	4.82	1.31	
		O–C=O	288.26	6.52	2.68	
		π-π*	290.62	4.71	3.26	
f-MWCNTs 200°C	**C1s**	C–C/C=C	284.77	58.40	1.33	7.48
		C–O/C=N	285.85	21.11	1.39	
		C=O/C–N	287.05	12.03	2.31	
		O–C=O	289.20	5.15	2.68	
		π-π*	291.43	3.31	3.26	
f-MWCNTs 300°C	**C1s**	C–C/C=C	284.77	61.22	1.33	14.37
		C–O/C=N	285.87	21.69	1.32	
		C=O/C–N	287.08	6.34	1.52	
		O–C=O	288.56	6.93	2.68	
		π-π*	291.06	3.81	3.26	

Table S2. The results of N1s core level lines fit pristine and calcined f-MWCNTs nanotubes.

Sample	Line	Component	Peak position [eV]	Area [%]	FWHM	χ-Squared
f-MWCNTs	N1s	N1	398.97	27.16	1.58	1.15
		N2	400.32	36.92	2.28	
		N3	401.97	25.25	2.31	
		N4	406.89	10.67	4.98	
f-MWCNTs 100°C	N1s	N1	398.73	34.35	1.53	1.49
		N2	400.21	52.16	2.15	
		N3	402.24	13.50	3.67	
f-MWCNTs 200°C	N1s	N1	398.72	35.79	1.53	1.56
		N2	400.30	46.98	2.08	
		N3	402.50	17.23	4.46	
f-MWCNTs 300°C	N1s	N1	398.68	34.99	1.53	1.83
		N2	400.27	55.94	2.14	
		N3	402.96	9.07	3.18	

Table S3. The results of O1s core level lines fit pristine and calcined f-MWCNTs nanotubes. .

Sample	Line	Component	Peak position [eV]	Area [%]	FWHM	χ-Squared
f-MWCNTs	O1s	C=O	531.78	47.16	1.65	1.14
		C–O/C-OH	533.32	48.64	2.07 2	
		O in H_2O	535.61	4.20	1.87	
f-MWCNTs 100°C	O1s	C=O	531.62	14.29	2.02	1.26
		C–O/C-OH	533.09	52.02	2.46	
		Si–O/O in H_2O	534.86	33.69	4.39	
f-MWCNTs 200°C	O1s	C=O	531.69	18.46	2.05	1.02
		C–O/C-OH	533.29	49.67	2.54	
		Si–O/O in H_2O	536.64	31.87	5.00	
f-MWCNTs 300°C	O1s	C=O	531.56	14.30	2.01	1.05
		C–O/C–OH	533.21	53.25	2.26	
		Si–O/O in H_2O	536.21	32.45	4.39	

Table S4. The results of S2p core level lines fit pristine and calcined f-MWCNTs nanotubes.

Sample	Line	Component	Peak position [eV]	Area [%]	FWHM	χ-Squared
f-MWCNTs	S2p	S–C $S2p_{3/2}$	163.87	32.65	1.56	1.31
		S–C $S2p_{1/2}$	165.05	16.33	2.11	
		S–O $S2p_{3/2}$	168.64	34.01	1.58	
		S–O $S2p_{1/2}$	169.82	17.01	1.82	
f-MWCNTs 100°C	S2p	S–C $S2p_{3/2}$	163.63	59.32	1.44	1.52
		S–C $S2p_{1/2}$	164.81	29.66	1.46	
		S–O $S2p_{3/2}$	168.48	7.34	1.52	
		S–O $S2p_{1/2}$	169.66	3.67	2.15	
f-MWCNTs 200°C	S2p	S–C $S2p_{3/2}$	163.72	55.56	1.60	1.90
		S–C $S2p_{1/2}$	164.90	27.78	1.59	
		S–O $S2p_{3/2}$	168.31	11.10	2.15	
		S–O $S2p_{1/2}$	169.49	5.55	2.15	
f-MWCNTs 300°C	S2p	S–C $S2p_{3/2}$	163.79	66.67	1.19	1.18
		S–C $S2p_{1/2}$	164.97	33.33	1.04	

Table S5. The results of Fe2p core level lines fit for f-MWCNTs nanotubes calcined at 200°C.

Sample	Line	Component	Peak position [eV]	Area [%]	FWHM	χ-Squared
f-MWCNT 200°C	Fe2p	Overlapped Fe/ Fe_3C (dominated by Fe_3C)	708.06	20.89	2.16	1.30
		α-FeOOH /γ-FeOOH	710.68	29.63	4.75	
		sat.	715.18	16.15	4.78	
		Overlapped Fe/ Fe_3C (dominated by Fe_3C)	721.16	10.44	2.77	
		α-FeOOH /γ-FeOOH	723.78	14.82	4.75	
		Sat.	728.28	8.08	5.08	
